# History of Medicine from the Earliest Ages to the Commencement of the Nineteenth Century

**Published:** 1872-06

**Authors:** 


*History of Medicine from the earliest ages to the commencement of the Nineteenth Century.* By ROBLEY DUNGLISON, M. D., LL. D. etc. Arranged and edited by Richard J. Dunglison, M. D. Philadelphia: Lindsay & Blakiston, 1872. (By subscription only.)

This very interesting book comprises the substance of a course of lectures delivered by the late Prof. Dunglison, while connected with the university of Virginia.

The history of medicine is gradually traced from the earliest ages, when the healing art was surrounded with superstition and the mysteries heathen religion and pagan sacrifice, down to the periods of discovery and research, when the medical profession to a greater or less extent threw aside the covering of tradition and superstitious awe, and allowed the clear light of scientific research to shine through the darkness which had hitherto covered all that pertained to the art of healing. The errors into which the leading minds of the past were so easily led by following a theory or fancy, which had taken possession of their minds, to the exclusion of all other facts, and which was blindly followed by the deluded teacher and his disciples, without once pausing to investigate the cause or effect of the natural phenomena, which they were daily observing, is forcibly illustrated, and should teach a lesson to some of the men of our time who delight to, what is called in common language "ride a Hobby."

The men and teaching of the past are brought before us and introduced to our attention in the concise, brief and vivid language for which Prof. Dunglison is so well noted. The reader can neither grumble because the writer dwells too long on uninteresting details, or because he too briefly touches the important points of his subject. To all readers this volume will be a treasure; serving as a condensed history to the practitioner who has not the time or inclination to read more extended works, and as a guide and incentive to further research to him who wishes to investigate more fully the details of medical history.

